# Draft genome sequence of the mammalian pathogenic *Paralagenidium karlingii*

**DOI:** 10.1128/mra.00442-26

**Published:** 2026-08-27

**Authors:** Maiara Goncalves Ramos, Fahong Yu, Rebecca Barber, Rebecca Nance-Richey, Jessica Gilbertie, Averi Kaplan-Hsu, Erica M. Goss, Domenico Santoro, Aline Rodrigues Hoffmann

**Affiliations:** 1 Department of Comparative, Diagnostic, and Population Medicine, College of Veterinary Medicine, University of Florida 3463, Gainesville, Florida, USA; 2 Interdisciplinary Center for Biotechnology Research, University of Florida 3463, Gainesville, Florida, USA; 3 Veterinary Diagnostic Laboratories, College of Veterinary Medicine, University of Florida 3463, Gainesville, Florida, USA; 4 Department of Small Animal Clinical Sciences, College of Veterinary Medicine, University of Florida 3463, Gainesville, Florida, USA; 5 Virginia Tech Animal Laboratory Services, Virginia Maryland College of Veterinary Medicine 70732, Blacksburg, Virginia, USA; 6 Veterinary Skin and Ear, Los Angeles, California, USA; 7 Department of Plant Pathology, Institute of Food and Agricultural Sciences and Emerging Pathogens Institute, University of Florida 3463, Gainesville, Florida, USA; Rochester Institute of Technology, Rochester, New York, USA

**Keywords:** oomycetes, pathogens, genomes, mammalian oomycetes, characterization

## Abstract

A draft genome sequence of the mammalian pathogenic oomycete *Paralagenidium karlingii* was generated from canine clinical isolate samples collected in the Eastern United States. Genome assembly was performed from a combination of long- and short-read sequencing data, followed by annotation of a 16,944 predicted protein-coding genes data set.

## ANNOUNCEMENT

*Paralagenidium karlingii* causes cutaneous and subcutaneous diseases, primarily in dogs (1, 2). Its genome was previously published by Hathcock et al. (3). Here, we report an additional genome sequence of *P. karlingii* to strengthen genomic characterization and support assessments of intraspecific diversity and species boundaries. Clinical samples from dogs in Virginia and Georgia, USA, were submitted to our facility for suspected oomycosis, and pure isolates were obtained from residual diagnostic culture material by subculturing actively growing mycelium from the original Columbia blood agar plates (35°C, 48–72 h) onto freshly prepared Columbia plates. Mycelium from each isolate was lyophilized, bead-homogenized, and gDNA extracted using the Wizard Genomic DNA Purification Kit (Promega) with the manufacturer’s protocol. DNA quality was assessed by Qubit, NanoDrop, and TapeStation. Sequencing used 99.8 ng and 469–520 ng gDNA, purity ratios 1.8 (260/280) and 2.0 (260/230), and fragments ≥ 60 kb. The internal transcribed spacer (ITS) and the large subunit (LSU) amplification confirmed *P. karlingii* identity, yielding 99.45% (KT257345.1) and 99.89% (KT257380.1) BLAST matches for the Virginia isolate and 100% identity (KT257345.1) for the Georgia isolate. Illumina sequencing was performed for the Virginia isolate, and both *P. karlingii* isolates underwent Nanopore sequencing. The Georgia isolate was sequenced twice to increase yield, generating three read sets that were pooled for assembly. Illumina MiSeq (2 × 250) library was prepared using the Illumina DNA Prep Kit and AMPure XP beads. Cutadapt (v3.4) (4) trimming produced 6,953,573 reads, and FastQC (v0.12.1) (5) generated quality metrics. Nanopore libraries were prepared using the PCR-free ONT Ligation Sequencing Kit (Oxford Nanopore Technologies, #SQK-NBD114.96) with the NEBNext Companion Module (New England Biolabs, #E7180L) and sequenced on an ONT GridION (R10.4.1 flow cell). Dorado (v0.5.3) basecalling used DNA super-accurate and 5mC/5hmC models, and demultiplexing used Dorado-demultiplex (SQK-NBD114.96). Samtools fastq (v1.20) (6) produced FASTQ files. Nanopore sequencing generated 2,026,423 reads (A), 684,401 (B), and 830,234 (C) (3,541,058 total) with estimated 71× coverage. Raw read *N*50 values were 3,976 bp (A), 1,400 bp (B), and 2,559 bp (C), averaging 2,645 bp. Combined reads yielded 3.59 Gb of sequence data. Porechop (v0.2.4) (7) filtered low-quality reads. A hybrid assembly integrating long and short reads for increased coverage (8) was performed using Flye (v2.9.3) (9) and Pilon (v1.24) (10), producing a 52,190,285-bp draft genome. Assembly and annotation were conducted with funannotate (v1.8.17) (11), including clean, mask, sort, predict (AUGUSTUS: *Tetrahymena thermophila*), and annotate modules, with InterProScan (v5.75-106.0) (12), EggNOG-mapper (v2.1.12) (13), QUAST (v5.3.0) (14), and BUSCO (v5.8.3; eukaryota_odb10) (15). Assembly and annotation statistics are shown in Table 1.

**TABLE 1:** Summary statistics for the *Paralagenidium karlingii* genome assembly and annotation

Characteristics	Value
Total assembly length (Mb)	52.1
No. of scaffolds	2,430
No. of contigs (≥1,000 bp)	1,520
No. of contigs (≥5,000 bp)	726
No. of contigs (≥10,000 bp)	588
Largest contig	975,956
N50 (bp)	160,888
L50	90
Total BUSCO groups	255
No. of complete BUSCOs (%)	84.3%
No. of complete single-copy BUSCOs (%)	83.1%
No. of duplicate single-copy BUSCOs (%)	1.2%
No. of incomplete BUSCOs (%)	4.7%
No. of missing BUSCOs (%)	11%
%GC	51
Total no. of predicted protein-coding genes	16,944
NCBI BioProject accession no.	PRJNA1334418
GenBank assembly accession no.	JBVVWT000000000

Putative virulence factors previously described in *Pythium insidiosum* (16–18) were detected in the predicted *P. karlingii* gene set, including urease, WD-40 repeat proteins, serine/threonine proteases, and cyclophilin A-like. Although *P. karlingii* is phylogenetically unrelated to *P. insidiosum* (2), these similarities may reflect parallel adaptation to mammalian infection. Elicitin and crinkler effectors, typically associated with plant-pathogenic oomycetes (19), were also present, indicating retention of ancestral gene families with potential functional adaptation to mammalian hosts.

## Data Availability

The related genome sequence of *Paralagenidium karlingii* has been deposited on National Center for Biotechnology Information (NCBI) under BioProject PRJNA1334418 and the accession number JBVVWT000000000. The raw sequencing reads have been deposited in the NCBI Sequence Read Archive under accession numbers SRR37707775 and SRR37707776.
